# Analysis of postoperative pulmonary complications after gastrectomy for gastric cancer: development and validation of a nomogram

**DOI:** 10.3389/fsurg.2023.1308591

**Published:** 2023-12-21

**Authors:** Ling Zhou, Yuanna Li, Yuanbo Ni, Cunming Liu

**Affiliations:** Department of Anesthesiology and Perioperative Medicine, The First Affiliated Hospital of Nanjing Medical University, Nanjing, Jiangsu, China

**Keywords:** gastrectomy, risk factors, nomogram, state of nutrition, complications

## Abstract

**Background:**

Postoperative pulmonary complications (PPCs) are common in gastric cancer patients after gastrectomy. The aim of our study was to investigate the perioperative risk factors and to develop a nomogram to identify patients who are at significant risk of PPCs.

**Methods:**

The clinical data of gastric cancer patients who underwent elective gastrectomy in the First Affiliated Hospital of Nanjing Medical University from 2017 to 2021 were retrospectively collected. All patients were randomly divided into a training and a validation cohort at a ratio of 7:3. Univariate and multivariate analysis were applied to identify the independent risk factors that might predict PPCs, and a nomogram was constructed. Both discrimination and calibration abilities were estimated by the area under a receiver operating characteristic curve (AUC) and calibration curves. The clinical effectiveness of the nomogram was further quantified with the decision curve analysis (DCA).

**Results:**

Of 2,124 included patients, one hundred and fifty patients (7.1%) developed PPCs. Binary logistic analysis showed that age > 65 years, higher total cholesterol level, longer duration of surgery, total gastrectomy, and the dose of oxycodone > 5.5 mg were independent risk factors for the occurrence of PPCs, which were contained in the nomogram. The predictive nomogram showed good discrimination and calibration [an AUC of 0.735 (95% CI: 0.687–0.783) in a training cohort and 0.781 (95% CI: 0.715–0.847) in a validation cohort]. The calibration curve and decision curve analysis showed a good agreement between nomogram predictions and actual observations.

**Conclusion:**

We developed a nomogram model based on age, total cholesterol, extent of resection, duration of surgery, and the dose of oxycodone to predict the risk of PPCs in gastric cancer patients after elective gastrectomy.

## 1. Introduction

Gastric cancer (GC) is one of the most common malignant tumors, and the third leading cause of cancer-related death throughout the world (1). It is highly prevalent in China (2). The incidence of GC increases in the elderly due to the aging process (3). Therapeutic radical gastrectomy is one of the major strategies to improve the prognosis of GC. Though the perioperative treatments, surgical skills and sophisticated anesthesiologic managements continue to be in progress, it is still difficult to avoid postoperative complications, especially after the upper-abdominal surgery. The incidence of postoperative complications is about 10%—50%, of which the incidence of postoperative pulmonary complications (PPCs) is relatively high (4, 5). Patients with severe PPCs may have to be admitted to intensive care unit (ICU) for further treatments, or even die. PPC is one of the major causes affecting the prognosis of patients and increasing medical costs (6). Therefore, seeking for an effective method to predict the complications as early as possible may reap even more benefits.

The occurrence of PPCs is related to multiple factors, including preoperative status of patients, surgical-related factors, and postoperative recovery. Chronic comorbidities, such as chronic obstructive pulmonary disease (COPD) or poor nutritional status, may increase the risk of PPCs (7, 8). Patients with GC often suffer from malnutrition, frailty, or cachexia, which has unfavorable effects on respiratory muscle mass and strength, thereby increase the risk of PPCs and other complications (9). In addition, surgical- and anesthesia- related factors, including surgery approaches, gastric resection methods, anesthesia management, and anesthetic drugs, also have an impact on the incidence of PPCs (10). The pain may arise due to the large incision after open surgery or the urinary catheters and drainage tubes after surgery, which further prevent the patients from respiratory training and delay the recovery of postoperative pulmonary function (11). Moreover, a randomized clinical trial reported that the incidence of PPCs was still as high as 38% after mechanical ventilation regardless of the ventilation strategy of low or high tidal volume with positive end expiratory pressure (PEEP) (12, 13). Analgesics are also a kind of risk factors when excessively administrated, which could lead to excessive sedation and respiratory depression (14).

Unfortunately, there are few relevant studies evaluating perioperative factors of patients to comprehensively prevent and recognize the incidence of overall PPCs after radical gastrectomy. Therefore, we conducted this study to explore the possible risk factors in the perioperative period and to establish a prediction model with a nomogram to identify patients at high risk of PPCs.

## 2. Materials & methods

### 2.1. Study design and ethical issues

This was a retrospective, observational, single-centered cohort study. All patients undergoing gastrectomy at the department of general surgery of The First Affiliated Hospital of Nanjing Medical University from January 2017 to December 2021 were retrospectively recruited in the study.

The inclusion criteria were (1) >18 years old; (2) diagnosed with gastric cancer; and (3) undergoing elective gastrectomy. Patients with incomplete clinical records or missing data were excluded.

The study was conducted in accordance with the Declaration of Helsinki. The protocol of this study was approved by the ethical committee of the First Affiliated Hospital of Nanjing Medical University (No: 2023-SR-224). Due to the retrospective study design, informed patient consent was waived.

### 2.2. Data collection and calculations

Clinical data were extracted from the electronic medical records, including age, gender, height, weight, body mass index (BMI), American Society of Anesthesiologists (ASA) score, chronic comorbidity [including hypertension, diabetes, cardiovascular diseases, chronic pulmonary disease (COPD), cerebrovascular accident history, renal diseases, smoking history and drinking history]. The laboratory parameters, including hemoglobin, albumin, total cholesterol, neutrophil count, lymphocyte count, and preoperative partial oxygen pressure at admission, were collected. Extent of resection, surgical approaches, duration of surgery, volume of intraoperative fluids, amount of bleeding, anesthetics, as well as analgesic managements were recorded and analyzed. Postoperative parameters including the length of postoperative hospital and discharge location were also gathered.

The nutritional status scores, including the control nutritional status score (CONUT), the geriatric nutritional risk index (GRNI), and the prognostic nutritional index (PNI) were calculated based on the relevant clinical data:

The CONUT score is calculated based on the results of serum albumin level, cholesterol level and total lymphocyte count (Supplementary Table 1) (15).
The PNI score = concentration of serum albumin (g/L) + 5 × total lymphocyte count (×10^9^/L) (16).
The GNRI score = 1.489 × concentration of serum albumin (g/L) + 41.7 × (current weight/ideal weight), and the ideal weight = 22 × height^2^ (m) (17).

### 2.3. Definition of PPCs

The PPCs in this study was a composite of the in-hospital events. The definition of PPCs was based on a previous study, which consisted of pneumonia, pleural effusion, atelectasis, pneumothorax, and respiratory failure (18).

### 2.4. Nomogram construction and evaluation

All patients were randomly assigned to training or validation cohorts based on a ratio of 7:3 (19). Potential risk factors related to PPCs were screened out with univariate analysis. And the factors with a *p* value < 0.05 were further analyzed with a binary logistic regression model to identify the independent predictors of PPCs. Then the independent predictors in the training cohort were utilized to build a nomogram to visualize the results.

The accuracy of the nomogram was assessed with the area under the receiver operating characteristic (ROC) curve (AUC, or c-statistic), and calibration plots in both training and validation cohort. We made *a priori* decision only when an AUC c-statistic of models ≥ 0.70. The decision curve analysis (DCA) was further conducted to assess the clinical usefulness of the new models.

### 2.5. Sample size calculation

A large cohort study reported the incidence rate of PPCs was 15% (20). About 21 factors were considered as risk factors in this study. So, the sample size could be 2,100 patients calculated according to events per variable (EPV) rule.

### 2.6. Statistical analysis

Continuous variables are expressed as mean (standard deviation) or median [interquartile ranges] depending on the distribution of the data, and categorical variables are expressed as number (percentage). Differences between patients with and without PPCs were analyzed using the *t*-test or Mann–Whitney *U* test for continuous variables, and *chi^2^* test or *Fisher's* exact test for categorical variables.

SPSS (version 23.0; IBM, Inc, Chicago, IL) was used to carry out the statistical analysis. Two-tail *p* < 0.05 was considered statistically significant. The R programming language (version R 4.3.1; R Foundation for Statistical Computing, Vienna, Austria) was used to perform a binary logistic regression analysis model for building the nomogram, ROC curves, Calibration curves, and DCA curves.

## 3. Results

**Figure 1:**
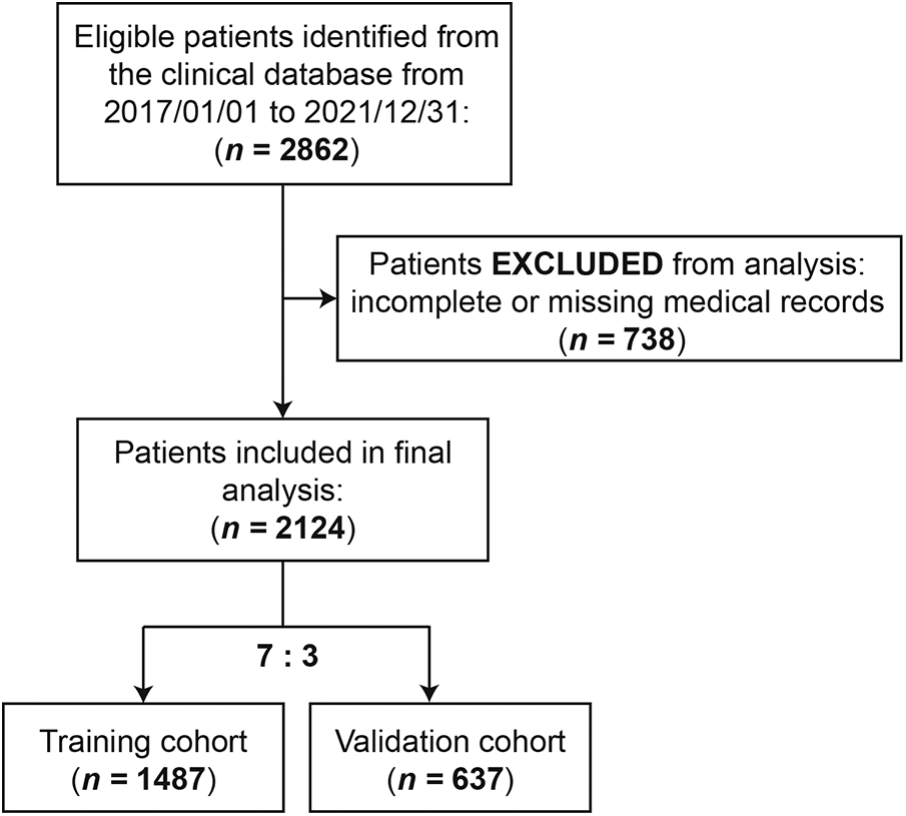
Flowchart of patients included for data analysis.

A total of 2,862 GC patients undergoing gastrectomy in the First Affiliated Hospital of Nanjing Medical University between 2017 and 2021 were identified. Seven hundred and thirty-eight patients were excluded due to incomplete or missing medical records. The demographic and clinical characteristics of 2,124 patients were eventually analyzed in this study (Figure 1).

### 3.1. Baseline characteristics and perioperative parameters

The baseline characteristics and perioperative parameters of all patients on admission are presented in Table 1. The number of patients over 65 years accounted for 41.20% in our study. A percentage of 72.60 were male. Among patients undergoing gastrectomy for GC, PPCs developed in one hundred and fifty (7.1%) patients. A larger proportion of old people (over 65 years) was observed in the PPCs group compared with the non-PPCs group [95 (63.33%) vs. 780 (39.51%), *p* < 0.001]. The patients with an ASA score of 3 and 4 accounted for 23.33% in the PPCs group, while the percentage was 15.25% in the non-PPCs group (*p* = 0.014).

**Table 1:** Baseline characteristics and perioperative parameters of gastric cancer participants.

Variables	Overall (*n* = 2,124)	non-PPCs (*n* = 1,974)	PPCs (*n* = 150)	*p*-value^a^	Training cohort (*n* = 1,487)	Validation cohort (*n* = 637)	*p*-value^b^
Demographics							
Age > 65 years (%)	875 (41.20)	780 (39.51)	95 (63.33)	<0.001	598 (40.21)	277 (43.49)	0.163
Male sex (%)	1,542 (72.60)	1,424 (72.14)	118 (78.67)	0.088	1,082 (72.76)	460 (72.21)	0.791
BMI (kg/m^2^)	23.56 (3.07)	23.55 (3.06)	23.79 (3.22)	0.361	23.51 (3.07)	23.69 (3.07)	0.239
ASA score (%)				0.014	** **	** **	0.517
1–2	1,788 (84.18)	1,673 (84.75)	115 (76.67)		1,257 (84.53)	531 (83.35)	
3–4	336 (15.82)	301 (15.25)	35 (23.33)		230 (15.47)	106 (16.64)	
Chronic comorbidity (%)							
Hypertension	576 (27.12)	529 (26.79)	47 (31.33)	0.253	401 (26.97)	175 (27.47)	0.831
Diabetes	184 (8.66)	173 (8.76)	11 (7.33)	0.652	122 (8.20)	62 (9.73)	0.274
Cardiovascular diseases	77 (3.63)	69 (3.50)	8 (5.33)	0.253	49 (3.30)	28 (4.40)	0.254
COPD	55 (2.59)	46 (2.33)	9 (6.00)	0.013	36 (2.42)	19 (3.00)	0.458
Cerebrovascular accident history	103 (4.85)	91 (4.61)	12 (8.00)	0.074	74 (4.98)	29 (4.60)	0.741
Renal diseases	23 (1.08)	21 (1.06)	2 (1.33)	0.675	17 (1.14)	6 (0.94)	0.821
Smoking history	606 (28.53)	566 (28.67)	40 (26.67)	0.640	418 (28.11)	188 (29.51)	0.529
Drinking history	430 (20.24)	406 (20.57)	24 (16.00)	0.206	288 (19.37)	142 (22.29)	0.126
Laboratory parameter							
Hemoglobin (g/L)	125.30 (22.84)	125.75 (22.63)	119.39 (24.72)	0.001	125.68 (22.86)	124.41 (22.77)	0.241
Albumin (g/L)	38.64 (4.42)	38.74 (4.42)	37.28 (4.24)	<0.001	38.75 (4.42)	38.37 (4.41)	0.072
Total cholesterol (mmol/L)	4.41 (1.00)	4.44 (1.00)	4.09 (1.04)	<0.001	4.41 (1.01)	4.41 (0.96)	0.956
Neutrophil count (*10^9^/L)	4.11 (2.61)	4.09 (2.62)	4.28 (2.55)	0.393	4.08 (2.58)	4.17 (2.69)	0.477
Lymphocyte count (*10^9^/L)	1.51 (0.58)	1.51 (0.58)	1.45 (0.57)	0.188	1.51 (0.57)	1.50 (0.59)	0.945
Preoperative partial oxygen pressure (mmHg)	90.19 (12.63)	90.26 (12.69)	89.29 (11.89)	0.368	90.03 (12.80)	90.56 (12.24)	0.382
Nutritional status							
CONUT	2.00 [1.00–3.00]	2.00 [1.00–3.00]	2.50 [1.00–4.00]	0.001	2.00 [1.00–3.00]	2.00 [1.00–3.00]	0.691
PNI	46.16 (5.85)	46.29 (5.85)	44.51 (5.61)	<0.001	46.28 (5.84)	45.89 (5.86)	0.164
GNRI	102.19 (9.44)	102.32 (9.41)	100.60 (9.71)	0.032	102.27 (9.43)	102.03 (9.46)	0.594
Surgical and anesthetic parameter							
Extent of resection (%)				<0.001	** **	** **	0.812
Partial gastrectomy	1,163 (54.76)	1,124 (56.94)	39 (26.00)		817 (54.94)	346 (54.32)	
Total gastrectomy	961 (45.24)	850 (43.06)	111 (74.00)		670 (45.06)	291 (45.68)	
Surgical approach (%)				0.003	** **	** **	0.493
Open surgery	593 (27.92)	535 (27.10)	58 (38.67)		422 (28.38)	171 (11.50)	
Laparoscopic surgery	1,531 (72.08)	1,439 (72.90)	92 (61.33)		1,065 (71.62)	466 (73.16)	
Duration of surgery (min)	180.28 (44.89)	179.05 (42.07)	196.39 (70.61)	0.003	179.69 (45.58)	181.64 (43.22)	0.356
Intraoperative fluids (ml)	2108.52 (216.20)	2105.62 (213.26)	2146.67 (249.47)	0.052	2105.92 (214.02)	2114.60 (221.25)	0.397
Bleeding (ml)	76.18 (36.38)	75.96 (35.81)	79.13 (43.24)	0.382	75.64 (35.89)	77.46 (37.49)	0.290
Sugammadex (%)	179 (8.43)	161 (8.16)	18 (12.00)	0.125	115 (7.73)	64 (10.05)	0.088
Dose of oxycodone > 5.5 mg (%)	434 (20.43)	382 (19.35)	52 (34.67)	<0.001	314 (21.12)	120 (18.84)	0.241
Dose of fentanyl (mg)	0.55 (0.10)	0.55 (0.10)	0.55 (0.09)	0.912	0.55 (0.10)	0.55 (0.10)	0.690
Multimodal analgesia (%)	2,098 (98.78)	1,950 (98.78)	148 (98.67)	0.705	1,472 (99.00)	626 (98.27)	0.196
Postoperative parameters							
Length of postoperative hospital stay (days)	9.00 [8.00–10.00]	8.00 [7.00–10.00]	16.00 [12.00–26.00]	<0.001	9.00 [8.00–11.00]	8.00 [8.00–10.00]	0.225
Discharge location (%)				<0.001	** **	** **	0.079
Clinical ward	2,099 (98.82)	1,958 (99.19)	141 (94.00)		1,474 (99.13)	625 (98.12)	
ICU stay	25 (1.18)	16 (0.81)	9 (6.00)		13 (0.87)	12 (1.88)	

Data are presented as mean (standard deviation), median [interquartile ranges] or number (percentage).

ASA score, American society of anesthesiologists score; BMI, body mass index; CONUT, control nutritional status score; COPD, chronic obstructive pulmonary disease; GNRI, geriatric nutritional risk index; ICU, intensive care unit; PNI, prognostic nutritional index; PPCs, postoperative pulmonary complications.

^a^ Comparisons between the non-PPCs group and the PPCs group.

^b^ Comparisons between the training cohort and the validation cohort.

There was a higher prevalence of COPD [9 (6.00%) vs. 46 (2.33%), *p* = 0.013] in the patients with PPCs. The nutritional status indicators, including the level of hemoglobin (119.39 ± 24.72 vs. 125.75 ± 22.63 g/L, *p* = 0.001), albumin (37.28 ± 4.24 vs. 38.74 ± 4.42 g/L, *p* < 0.001), and total cholesterol (4.09 ± 1.04 vs. 4.44 ± 1.00 mmol/L, *p* < 0.001), were lower in patients suffering from PPCs. Meanwhile, the nutritional status scores (CONUT, PNI, and GNRI) were worse in the patients with PPCs (all *p* < 0.05) (Table 1). Patients were more likely to develop PPCs with total gastrectomy [111 (74.00%) vs*.* 39 (26.00%), *p* < 0.001], open surgery [92 (61.33%) vs*.* 58 (38.67%), *p* = 0.003] and longer duration of surgery (196.39 ± 70.61 vs*.* 179.05 ± 42.07 min, *p* = 0.003). Surprisingly, the analgesic, oxycodone, statistically increased the occurrence of PPCs. To determine the risk stratification classification for PPCs, the best cutoff value for oxycodone (5.5 mg) was calculated through the ROC curve. The dose of oxycodone > 5.5 mg significantly increased the occurrence of PPCs (*p* < 0.001) (Table 1). The median length of postoperative hospital stay was longer in patients with PPCs [16 (12–26) days] than those without PPCs [8 (7–10) days]. A higher probability of ICU stay was observed in patients with PPCs than those without PPCs [9 (6.00%) vs. 16 (0.81%), *p* < 0.001] (Table 1).

### 3.2. Training cohort and validation cohort

All patients were divided into a training cohort (*n* = 1,487) and a validation cohort (*n* = 637) in accordance with the random number table. Table 1 presented a detailed comparison of the predictive variables between the training and validation cohorts. There were no significant differences between the training cohort and the validation cohort (Table 1).

### 3.3. Univariate and multivariate predictors for PPCs

The univariate analysis was conducted as shown in Table 2. Univariate analysis revealed that age > 65 years, ASA score, level of hemoglobin, albumin, and total cholesterol, total gastrectomy, open surgery, duration of surgery, and the dose of oxycodone > 5.5 mg were significantly related to PPCs. The binary logistic regression analysis both in the training cohort and validation cohort showed that age > 65 years, lower level of total cholesterol, total gastrectomy, longer surgery duration, and the dose of oxycodone > 5.5 mg were independent risk factors for PPCs in patients after gastrectomy (Table 3).

**Table 2:** Results of univariate analysis in the training cohort.

Variables	non-PPCs (*n* = 1,378)	PPCs (*n *= 109)	*p*-value
Age > 65 years (%)	530 (38.46)	68 (62.39)	<0.001
ASA score 3–4 (%)	204 (14.80)	26 (23.85)	0.018
COPD (%)	31 (2.25)	5 (4.59)	0.180
Hemoglobin (g/L)	126.23 (22.59)	118.67 (25.14)	0.001
Albumin (g/L)	38.85 (4.41)	37.36 (4.40)	0.002
Total cholesterol (mmol/L)	4.43 (1.00)	4.09 (1.05)	0.001
CONUT	2 [1–3]	3 [1.5–4]	0.002
PNI	46.41 (5.84)	44.60 (5.61)	0.002
GNRI	102.39 (9.33)	100.64 (10.50)	0.062
Extent of resection (%)			<0.001
Partial gastrectomy	787 (57.11)	30 (27.52)	
Total gastrectomy	591 (42.89)	79 (72.48)	
Surgical approach (%)			0.003
Open surgery	377 (27.36)	45 (41.28)	
Laparoscopic surgery	1,001 (72.64)	64 (58.72)	
Duration of surgery (min)	178.42 (41.72)	191.12 (49.29)	0.010
Dose of oxycodone > 5.5 mg (%)	277 (20.10)	37 (33.94)	0.001

Data are presented as mean (standard deviation), median [interquartile ranges] or number (percentage).

ASA score, American society of anesthesiologists score; CONUT, control nutritional status score; COPD, chronic obstructive pulmonary disease; GNRI, geriatric nutritional risk index; PNI, prognostic nutritional index; PPCs, postoperative pulmonary complications.

**Table 3:** Significant variables for prediction of PPCs in the training and validation cohort via binary logistics regression analysis.

Variables	Subgroup	Training cohort (*n* = 1,487)			Validation cohort (*n* = 637)		
		OR	95% *CI*	*p*-value	OR	95% *CI*	*p*-value
Age (years)	≤65	1			1		
	>65	2.17	1.43–3.28	<0.001	2.24	1.12–4.51	0.023
Total cholesterol (mmol/L)		0.75	0.60–0.92	0.007	0.62	0.42–0.90	0.012
Extent of resection	Partial gastrectomy	1			1		
	Total gastrectomy	2.95	1.89–4.59	<0.001	3.72	1.72–8.08	0.001
Duration of surgery (min)		1.01	1.00–1.01	0.048	1.01	1.00–1.02	0.032
Dose of oxycodone (mg)	≤5.5	1			1		
	>5.5	1.85	1.20–2.84	0.005	2.67	1.31–5.42	0.007

CI, confidence interval; OR, odds ratio; PPCs, postoperative pulmonary complications.

### 3.4. Construction of the nomogram and validation

**Figure 2:**
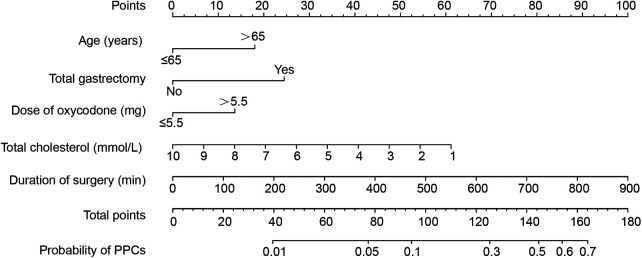
A nomogram for prediction of postoperative pulmonary complications in gastric cancer patients after elective gastrectomy.

**Figure 3:**
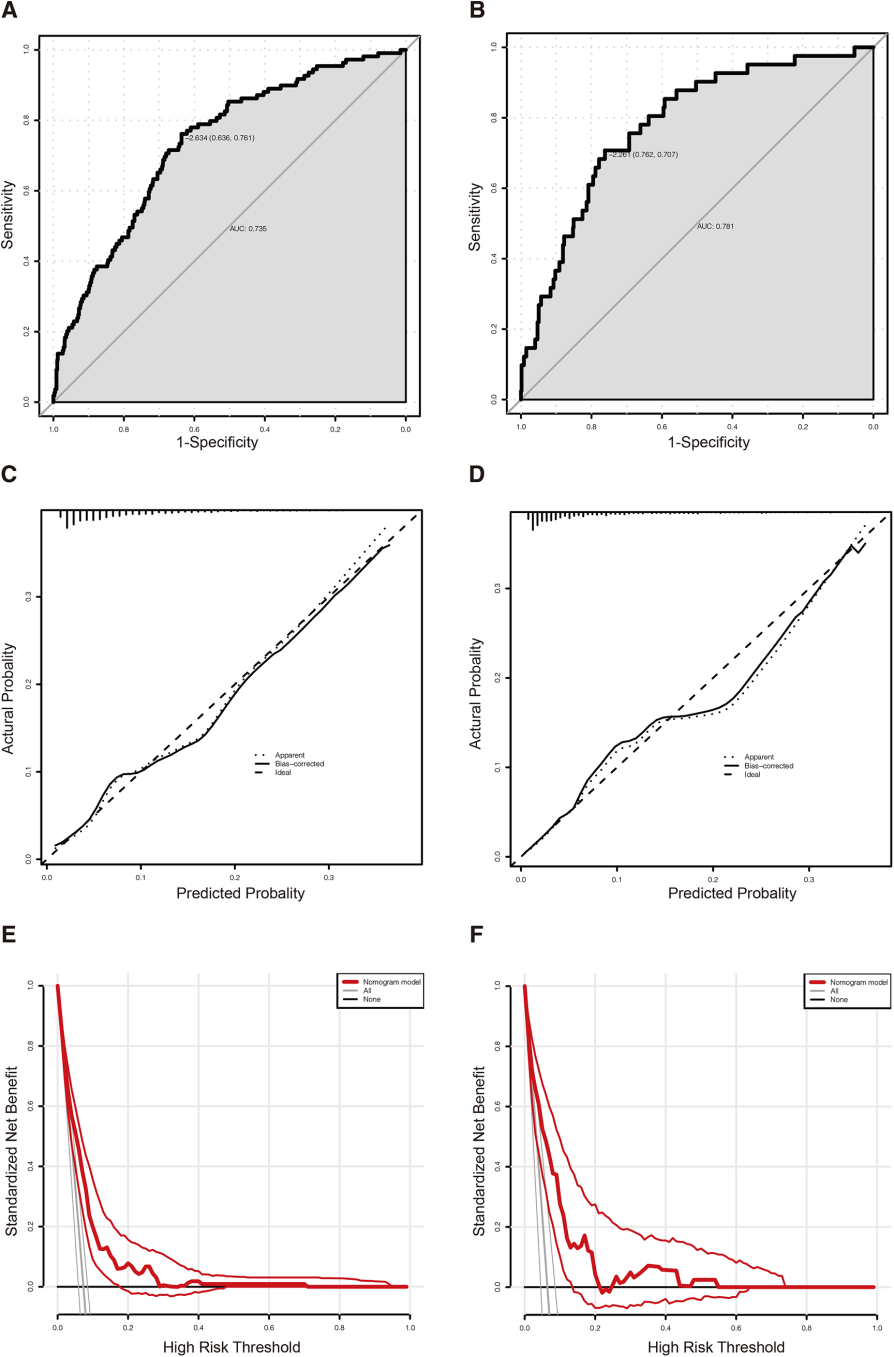
(**A**) The ROC curve of the predictive nomogram in the training cohort with an AUC value of 0.735 (95% CI: 0.687–0.783). (**B**) The ROC curve of the predictive nomogram in the validation cohort with an AUC value of 0.781 (95% CI: 0.715–0.847). (**C**) The calibration plot of the predictive nomogram in the training cohort. (**D**) The calibration plot of the predictive nomogram in the validation cohort. (**E**) The DCA curve of the predictive nomogram in the training cohort. (**F**) The DCA curve of the predictive nomogram in the validation cohort. ROC, receiver operating characteristic curve; DCA, decision curve analysis.

The nomogram of predictors was presented in Figure 2, which was constructed with five independent risk factors for PPCs in the binary logistic regression analysis. The nomogram model showed good discrimination and good calibration [an AUC of 0.735 (95% CI: 0.687–0.783) in the training cohort and 0.781 (95% CI: 0.715–0.847) in the validation cohort] (Figures 3A,B). The actual observations of probability and the calibration curves were shown in Figures 3C–F, which suggested a good agreement in both training cohort and validation cohort.

## 4. Discussion

In our study, 21 perioperative parameters were taken into consideration as predictors of PPCs for gastric cancer patients after elective radical gastrectomy, and 13 factors were identified by univariate analysis. The binary logistic regression analysis showed that age > 65 years, together with lower level of total cholesterol, total gastrectomy, longer duration of surgery, and the dose of oxycodone > 5.5 mg, were good predictors of PPCs. Hence, the nomogram was constructed. The AUC was 0.735 (95% CI: 0.687–0.783) in the training cohort and 0.781 (95% CI: 0.715–0.847) in the validation cohort.

Gastric cancer is one of the most malignant tumors and radical gastrectomy is associated with considerable morbidity, specifically PPCs (21). This study revealed that PPCs not only prolonged the postoperative recovery of patients and increased the postoperative hospital stay, but also increased the possibility of admission to the ICU, which were in consistent with previous studies (22, 23). The higher medical expenses and morbidity in PPCs patients arouse the attention to identify and distinguish the patients in high risks and make integrated plans to reduce the occurrence of PPCs. To this end, we designed this retrospective cohort study to seek for the predictors and develop a nomogram model to provide the clinicians with a visual tool to identify the high-risk patients and reduce the incidence of PPCs.

The incidence of PPCs increased with the prevalence of several independent risk factors, particularly advanced age, in this study. We found that patients over 65 years old have higher odds ratio of encountering with PPCs. The results of some clinical studies are similar with our study, as PPCs after gastrectomy may be age-related (10). This observation could be explained by the normal changes with aging. Natural lung aging caused the alterations in lung function, and increased susceptibility to chronic lung diseases (24). In our study, COPD was considered as a risk factor of PPCs in the univariate analysis, but it showed no significant difference in the logistic regression. However, some previous studies reported that preoperative existing diseases should be taken into consideration when evaluating the timing of surgery (25, 26). Avoiding performing the surgery in the acute episode of COPD is prerequisite in consideration of the rapid recovery of GC patients after surgery. Moreover, with aging, the number of patients receiving surgical treatment for GC is increasing (27). Critical care units are unavoidably needed if the patient is critically ill after surgery, which is a great challenge for the medical system.

To the best of our knowledge, nutritional status is another important indicator for postoperative recovery. The level of hemoglobin, albumin, and total cholesterol are some of the nutritional indicators (28). Total cholesterol was considered to be an important element to predict PPCs in this study. Delgado-Rodríguez et al. (29) believed the level of total cholesterol was a U-shaped relationship with postoperative respiratory tract infection. While Canturk et al. (30) reported that lower total cholesterol level (≤5.18 mmol/L) might be associated with nosocomial infections in surgical patients, which came to the similar conclusion with our study. The nutritious status, evaluated with CONUT, PNI and GNRI index, were in expectation of predicting prognosis. Lee et al. (31) recommends CONUT index as a good predictor of PPCs. In this study, however, despite the result of three nutritional status ranking system showed statistical differences between the non-PPCs group and PPCs groups. Multivariate analysis cannot prove a good sensitivity and specificity of the nutritious status between the groups. Therefore, we eventually included the potential risk factors according to the results of multivariate analysis and established a nomogram model.

The surgical approach, extent of resection and reconstruction depend on the lesion location, tumor size, and lymph node metastasis are suspicious characteristics in PPCs. In our study, the incidence of PPCs in open surgery for total gastrectomy was much higher than laparoscopic surgery for partial gastrectomy. The possible reason may be that the operation area is close to the diaphragm during total gastrectomy, and it could be stimulated and lead to PPCs, such as pleural effusion and atelectasis (32). Shin et al. (33) reported that surgery process had little association with postoperative complications. However, Lee et al. (31) argued that total gastrectomy was the independent risk factor for PPCs, which was in consistence with our study. As was reported, longer duration of surgery, as well as the longer period of mechanical ventilation, may lead to PPCs (34). Unfortunately, different ventilation strategies, with low- or high-volume tide, did not reduce the incidence of PPCs. And the strategy with additional PEEP had a slight effect on avoiding PPCs. Therefore, exploring a better surgical resection method may probably shorten the duration of mechanical ventilation and surgery, and therefore decrease PPCs.

Opioid use in the perioperative period is common for perioperative pain management. Sayal et al. (35) demonstrated that patients with opioid use disorders were at increased risk for PPCs. The increasing dose of opioids might lead to worse surgical outcomes and higher lung complications (36). There was little difference in the dose of fentanyl between PPCs group and non-PPCs group in our cohort study, but the dose of oxycodone was significantly different. A single dose of intravenous oxycodone > 5.5 mg before the abdomen closure was an independent risk factor for PPCs in this study. While oxycodone was reported to regulate inflammatory cytokines and reduce more inflammatory response to alleviate postoperative pain compared with sufentanil (37). Low dose of oxycodone should be recommended after gastrectomy.

Some limitations still exist. Firstly, this is a single-center retrospective study, which may prevent the applicability of the model. A prospective study should be carried out for external evidence for this prediction model. Secondly, some studies suggested PEEP improved outcomes for patients after surgery (38). However, we did not include PEEP as a potential factor as ventilation parameters were not recorded in the anesthesia note in our hospital, and PEEP was commonly set among GC patients during the surgery.

## 5. Conclusion

We developed a nomogram model based on age, total cholesterol, extent of resection, duration of surgery, and the dose of oxycodone to predict the probability of PPCs in GC patients after elective gastrectomy. The model may provide convenience for the surgeons to predict the risk of patients developing PPCs.

## Data Availability

The raw data supporting the conclusions of this article will be made available by the authors, without undue reservation.
