# Supplementary material for: Analysis of postoperative pulmonary complications after gastrectomy for gastric cancer: development and validation of a nomogram
Source: Front Surg. 2023 Dec 21;10:1308591. doi: 10.3389/fsurg.2023.1308591 (PMC10768169; doi:10.3389/fsurg.2023.1308591)
Supplement: Supplementary file 1 [file Table1.docx]

Supplementary Material

Analysis of postoperative pulmonary complications after gastrectomy for gastric cancer: development and validation of a nomogram

**Ling Zhou^1†^, Yuanna Li^1†^, Yuanbo Ni^1^, Cunming Liu^1^****^*^**

^1^ Department of Anesthesiology and Perioperative Medicine, The First Affiliated Hospital of Nanjing Medical University, Nanjing, Jiangsu Province, China

^†^ These authors contributed equally to this work and share first authorship.

*** Correspondence:**Cunming Liu
cunmingliu@njmu.edu.cn

**Supplementary Table 1.** Definition of CONUT

| Parameters | CONUT | | | |
| --- | --- | --- | --- | --- |
|  | Normal | Light | Moderate | Severe |
| Serum albumin (g/dL) | 3.5–4.5 | 3.0–3.49 | 2.5–2.9 | <2.5 |
| Alb score | 1 | 2 | 4 | 6 |
| Total lymphocyte (count/mm^3^) | ≥1600 | 1200–1599 | 800–1199 | <800 |
| TLC score | 0 | 1 | 2 | 3 |
| Total cholesterol (mg/dL) | >180 | 140–180 | 100–139 | <100 |
| TC score | 0 | 1 | 2 | 3 |
| CONUT score (total) | 0–1 | 2–4 | 5–8 | 9–12 |
| Assessment | Normal | Light | Moderate | Severe |

CONUT is calculated as the sum of the Alb score, TLC score, and TC score.

Alb, albumin; TLC, total lymphocytes; TC, total cholesterol.

Albumin (g/dL) = albumin (g/L) / 10; total lymphocyte (count/mm^3^) = total lymphocyte (10^^9^/L) × 1000; total cholesterol (mg/dL) = total cholesterol (mmol/L) × 38.67.
